# Impact of Resuscitative Endovascular Balloon Occlusion of the Aorta on In-Hospital and Short-Term Mortality: A Systematic Review and Meta-Analysis

**DOI:** 10.3390/diseases14040122

**Published:** 2026-03-27

**Authors:** Hiroyuki Kamide, Shingo Kato, Naofumi Yasuda, Shungo Sawamura, Yoshinobu Ishiwata, Nobuyuki Horita, Ryusuke Sekii, Tomohiro Oshima, Zenjiro Sekikawa, Daisuke Utsunomiya

**Affiliations:** 1 Department of Diagnostic Radiology, Yokohama City University Medical Center, Yokohama 232-0024, Japan; kamide.rad@gmail.com (H.K.); tauko@yokohama-cu.ac.jp (Z.S.); 2 Department of Diagnostic Radiology, Yokohama City University Graduate School of Medicine, Yokohama 236-0004, Japan; yasuda.nao.mz@yokohama-cu.ac.jp (N.Y.); sawa0808@yokohama-cu.ac.jp (S.S.); ishi_y@yokohama-cu.ac.jp (Y.I.); d_utsuno@yokohama-cu.ac.jp (D.U.); 3 Chemotherapy Center, Yokohama City University Hospital, Yokohama 236-0004, Japan; horitano@yokohama-cu.ac.jp; 4 Department of Internal Medicine, North Alabama Medical Center, Florence, AL 35630, USA; ryusuke.sekii@namccares.com; 5 Department of Diagnostic Radiology, Yokosuka General Medical Center, Yokosuka 239-8567, Japan; o.tomo.ohshima@gmail.com

**Keywords:** resuscitative endovascular balloon occlusion of the aorta (REBOA), mortality, trauma, geographic variations, systematic review and meta-analysis

## Abstract

Background: Resuscitative endovascular balloon occlusion of the aorta (REBOA) is increasingly employed in patients with hemorrhagic shock and cardiovascular collapse; however, its impact on mortality remains controversial. Differences in geographic regions and patient populations may influence clinical outcomes. Methods: We conducted a systematic review and meta-analysis of observational studies comparing mortality between patients receiving REBOA and those managed without REBOA. Pooled odds ratios (ORs) and 95% confidence intervals (CIs) were calculated using random-effects models. Subgroup analyses were performed according to propensity score (PS) matching, trauma versus non-trauma populations, and geographic regions. Results: A total of 10 studies involving 18,611 patients were included. Overall, REBOA was not associated with a significant reduction in mortality compared with non-REBOA (pooled OR = 0.52, 95% CI: 0.19–1.39, *p* = 0.19). In PS-matched studies, the pooled OR was 0.82 (95% CI: 0.34–1.98, *p* = 0.66), whereas in non-PS-matched studies it was 0.40 (95% CI: 0.12–1.26, *p* = 0.12). Geographic analyses revealed no significant mortality benefit in either Western studies (OR = 0.47, 95% CI: 0.12–1.89; *p* = 0.29) or non-Western studies (OR = 0.60, 95% CI: 0.11–3.38; *p* = 0.56). No survival benefit was observed among trauma patients (OR = 0.57, 95% CI: 0.20–1.61; *p* = 0.29), whereas a significant reduction in mortality was observed in non-trauma patients (OR = 0.21, 95% CI: 0.05–0.88; *p* = 0.03). Conclusions: In this systematic review and meta-analysis, REBOA was not associated with a significant reduction in mortality in the overall population or in trauma patients. However, in a single small non-trauma study (n = 53), REBOA was associated with significantly reduced mortality; this finding is exploratory and requires confirmation in larger prospective studies. These findings suggest that the clinical benefit of REBOA may depend on patient population and underlying etiology of hemorrhage.

## 1. Introduction

Non-compressible torso hemorrhage (NCTH) remains a leading cause of death in both traumatic and non-traumatic conditions, and its management continues to be a significant challenge in emergency medicine. Historically, resuscitative thoracotomy with aortic cross-clamping (RT-ACC) has been performed as a last resuscitative measure for patients nearing cardiac arrest. However, RT-ACC is extremely invasive, associated with severe physiological stress, and has poor survival and neurological outcomes, limiting its application [1,2]. This background has necessitated the urgent development of less invasive and more effective methods for maintaining hemodynamic stability and achieving hemostasis.

In recent years, Resuscitative Endovascular Balloon Occlusion of the Aorta (REBOA) has garnered attention as a minimally invasive alternative to RT-ACC. REBOA is an endovascular procedure in which a catheter is inserted percutaneously through the femoral artery, and a balloon is inflated within the aorta to maintain central blood pressure and control distal hemorrhage. Due to its advantages, such as being less invasive by avoiding the need for a thoracotomy and being feasible outside the operating room in settings like the emergency department, REBOA has been rapidly adopted by medical facilities worldwide. Early reports from large observational studies, such as the American Association for the Surgery of Trauma (AAST) AORTA registry, suggested the utility of REBOA and supported its widespread use [3]. However, despite its proliferation, the effect of REBOA on improving mortality remains a subject of significant debate. Some observational studies using propensity score matching have reported that REBOA is associated with reduced mortality [4,5]. Conversely, other studies with similar designs have suggested that REBOA might be harmful in patients with severe torso trauma [6], and an analysis using a U.S. nationwide database found no survival benefit [7], indicating a lack of consistent evidence.

This inconsistency in findings between studies, or heterogeneity, may be attributable to differences in the patient populations studied and the healthcare systems in which the treatment is provided. First, the effectiveness of REBOA may vary significantly depending on the underlying pathology causing the hemorrhage. For instance, the systemic physiological impact and therapeutic effects of REBOA are presumed to differ between trauma patients, who often have multiple injuries including head trauma, and non-trauma patients with more localized bleeding sources, such as ruptured abdominal aortic aneurysms (rAAA) or obstetric hemorrhage. Supporting this, the UK-REBOA trial, a recent randomized controlled trial considered to be the highest quality evidence to date, showed that REBOA did not improve 90-day mortality compared to standard care in patients with traumatic hemorrhagic shock [8]. However, several methodological considerations should be noted when interpreting these results. In that trial, the median time from emergency department admission to balloon inflation exceeded 30 min, and only a limited proportion of patients randomized to the REBOA group ultimately received successful catheter placement. These factors may have attenuated the potential clinical benefit of REBOA. In contrast, a study targeting patients with rAAA reported that REBOA might improve outcomes [9]. Second, geographic factors may also influence the results. Studies from Japan have frequently reported positive outcomes [10,11,12], whereas large-scale studies from Western countries have tended to show negative results. This could reflect differences in regional healthcare systems, including time from injury to treatment initiation, the proportion of blunt versus penetrating trauma, provider proficiency, and treatment protocols incorporating REBOA [13].

The results of individual studies published to date are conflicting, and a consensus on the optimal indications for REBOA has not been reached in the clinical setting. To resolve this clinical uncertainty, it is essential to synthesize the existing evidence and systematically evaluate the sources of heterogeneity in the results. Therefore, we conducted a systematic review and meta-analysis to comprehensively evaluate the effect of REBOA on mortality and to elucidate differences in its effectiveness through subgroup analyses based on patient population (trauma vs. non-trauma) and geographic region (Western vs. non-Western). The present review extends prior meta-analyses by incorporating studies published through October 2025, including the landmark UK-REBOA randomized controlled trial and multiple 2024–2025 publications. The present review additionally examines novel subgroup analyses by trauma versus non-trauma populations and geographic region, while systematically excluding overlapping cohorts from the same registries to avoid double-counting of cases.

## 2. Materials and Method

### 2.1. Eligibility Criteria and Literature Search Strategy

We conducted a comprehensive and systematic literature search to identify studies evaluating the impact of resuscitative endovascular balloon occlusion of the aorta (REBOA) on patient mortality. This systematic review and meta-analysis was conducted in accordance with the Preferred Reporting Items for Systematic Reviews and Meta-Analyses (PRISMA) 2020 guidelines. The search was performed using three major electronic databases: PubMed, Web of Science, and the Cochrane Library. All eligible articles published up to 27 October 2025, were considered for inclusion. The search strategy combined relevant Medical Subject Headings (MeSH) terms and free-text keywords, including “REBOA,” “resuscitative endovascular balloon occlusion of the aorta,” “mortality,” “trauma,” “non-trauma,” and “resuscitation.” The detailed search strategy for each database is provided in Supplemental Material 1. No restrictions were imposed on study design at the search stage to ensure comprehensive retrieval of potentially relevant studies. In addition to the electronic database search, the reference lists of all included articles and relevant review papers were manually screened to identify additional studies that may not have been captured through the initial database search. The study protocol was prospectively registered in the University Hospital Medical Information Network (UMIN) Clinical Trials Registry under the identifier UMIN000059701, ensuring methodological transparency and adherence to predefined study objectives.

### 2.2. Study Selection

Studies were considered eligible for inclusion if they met the following predefined criteria: (1) randomized controlled trials, prospective cohort studies, or retrospective observational studies; (2) studies that directly compared clinical outcomes between patients managed with resuscitative endovascular balloon occlusion of the aorta (REBOA) and those managed without REBOA; (3) studies that reported mortality as a primary or secondary outcome measure; and (4) studies that provided sufficient quantitative data to allow calculation of odds ratios (ORs) with corresponding 95% confidence intervals (CIs). Studies were excluded if they were animal or experimental studies, lacked an appropriate non-REBOA comparison group, did not report mortality outcomes, or failed to provide adequate data for effect size estimation. Studies involving overlapping or duplicate patient populations were also excluded to avoid double counting of cases. When multiple publications were identified from the same or overlapping datasets, only the most comprehensive report or the most recent publication with the largest sample size and most complete outcome data was included in the final analysis.

### 2.3. Data Extraction and Synthesis Methods

Two independent reviewers systematically extracted data from all eligible studies using a predefined data extraction form. Extracted variables included the first author’s name, year of publication, country of origin, study design, and key patient characteristics, with particular attention to whether the study population consisted of trauma or non-trauma patients. Additional data collected comprised sample size, specific indications for resuscitative endovascular balloon occlusion of the aorta (REBOA), and reported mortality outcomes. Any discrepancies or disagreements between the two reviewers during the data extraction process were resolved through discussion, and consensus was reached in all cases. The primary outcome of interest was all-cause mortality, defined as mortality assessed at the longest follow-up period reported in each individual study. Prespecified subgroup analyses were conducted to explore potential effect modification and sources of heterogeneity. These subgroup analyses stratified studies according to trauma versus non-trauma populations, Western versus non-Western geographic regions, and cohorts analyzed using propensity score (PS)–matched methods versus those without PS matching. In studies reporting propensity score–matched analyses, matching was performed using baseline variables related to injury severity and physiological status as reported in the original studies. These variables commonly included age, sex, injury severity score (ISS), mechanism of injury, systolic blood pressure on admission, Glasgow Coma Scale score, and the presence of traumatic cardiac arrest. When propensity score–matched cohorts were available, the matched populations were preferentially extracted for pooled analyses.

### 2.4. Risk of Bias Assessment

The methodological quality of the included observational studies was independently assessed using the Newcastle–Ottawa Scale (NOS), which evaluates study quality across three predefined domains: selection of study groups, comparability of cohorts, and ascertainment of outcomes. Each study was assigned a total score ranging from 0 to 9 points based on the NOS criteria. In accordance with established conventions, studies scoring 7–9 points were classified as high quality, those scoring 4–6 points as moderate quality, and those scoring ≤3 points as low quality. To evaluate the presence of potential publication bias, funnel plots were constructed by plotting individual study effect estimates against their corresponding standard errors. These plots were visually inspected for asymmetry, which may suggest the presence of small-study effects or selective publication.

### 2.5. Statistical Analysis

Pooled odds ratios (ORs) with corresponding 95% confidence intervals (CIs) were calculated to quantify the overall effect estimates across studies. A random-effects model based on the inverse variance weighting method was applied, taking into account both within-study and between-study variability and assuming that the true effect size may vary among individual studies. Statistical heterogeneity across the included studies was systematically evaluated using the I^2^ statistic, which describes the proportion of total variability attributable to between-study heterogeneity rather than chance. Consistent with commonly accepted criteria, I^2^ values of <25%, 25–50%, and >50% were interpreted as indicating low, moderate, and high heterogeneity, respectively. Prespecified subgroup analyses were conducted to explore potential sources of heterogeneity and to assess the robustness of the pooled estimates. These subgroup analyses compared outcomes between trauma-related and non-trauma cohorts, as well as between studies conducted in Western and non-Western geographic regions. Potential publication bias was initially assessed through visual inspection of funnel plot symmetry. In addition, Egger’s regression test was performed to provide a quantitative assessment of small-study effects and funnel plot asymmetry. All statistical analyses were performed using Review Manager (RevMan, Version 5.4; The Cochrane Collaboration, 2020) and R software ver 5.4 (R Foundation for Statistical Computing, Vienna, Austria). A two-sided *p*-value of less than 0.05 was considered to indicate statistical significance.

## 3. Results

### 3.1. Study Characteristics

**Figure 1:**
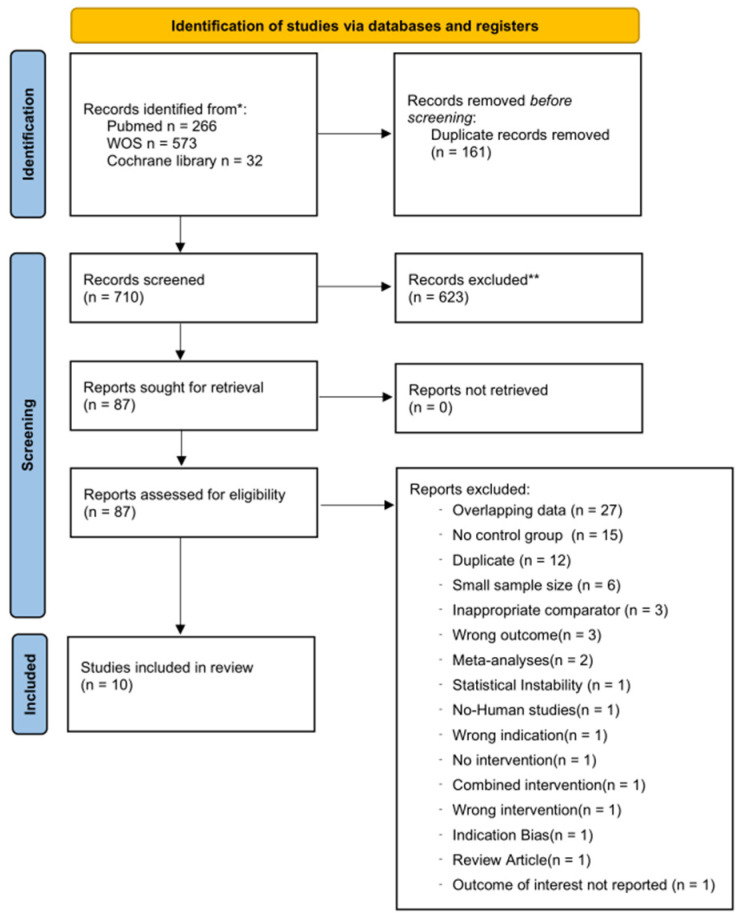
PRISMA flow diagram. These symbols in figure have no specific meaning.

The process of study identification, screening, eligibility assessment, and inclusion is illustrated in the PRISMA flow diagram (Figure 1). Table 1 summarizes the characteristics of the included studies. Among the 10 studies, nine were retrospective observational studies [1,7,9,10,14,15,16,17,18] and one was a randomized controlled trial [8]. Most studies focused on trauma populations (9 studies) [1,7,8,10,14,15,16,17,18], while one study evaluated non-traumatic hemorrhage due to ruptured abdominal aortic aneurysm [9]. Propensity score matching was applied in five of the nine retrospective studies (55.6%) [7,10,14,16,17], corresponding to 50% of all included studies. Patient age was reported in seven studies (70%) [1,7,9,14,15,16,17], with median or mean ages generally ranging from the early 30s to the early 70s, indicating predominantly middle-aged to older adult populations. Sex distribution was reported in six studies (60%) [1,7,9,14,16,17], with female patients accounting for approximately 13% to 34% of study populations.

### 3.2. Results of Risk of Bias Assessment

According to the NOS, a total of nine observational studies were assessed for methodological quality. Five studies (55.6%) were judged to be of high quality (NOS score 7–9), while four studies (44.4%) were classified as moderate quality (NOS score 4–6). No study was rated as low quality (NOS score ≤3) (Supplemental Material 2). Visual inspection of the funnel plot demonstrated asymmetry, suggesting potential small-study effects or publication bias, which was supported by Egger’s regression test (z = 2.17, *p* = 0.03) (Supplemental Material 3).

### 3.3. Overall Mortality Comparison Between REBOA and Non-REBOA

**Figure 2:**
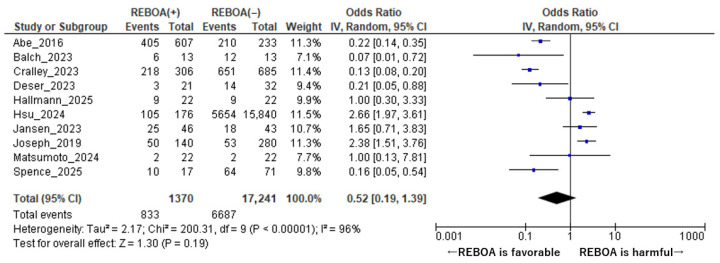
Forest Plot of Mortality Comparing REBOA and Non-REBOA Groups. The forest plot was constructed using data from references [1,7,8,9,10,14,15,16,17,18].

A total of 10 studies comprising 18,611 patients were included in the final meta-analysis, of whom 1748 patients were managed with REBOA and 17,959 patients received non-REBOA management [1,7,8,9,10,14,15,16,17,18]. These studies encompassed a wide range of clinical settings and patient populations, reflecting both traumatic and non-traumatic causes of hemorrhagic shock. In the overall pooled analysis, the use of REBOA was not associated with a statistically significant reduction in mortality when compared with non-REBOA management. The pooled odds ratio for mortality was 0.52 (95% CI: 0.19–1.39, *p* = 0.19), indicating no clear survival advantage for REBOA in the combined study population (Figure 2). Although the point estimate favored REBOA, the wide confidence interval crossing unity suggests substantial uncertainty around the magnitude and direction of the effect. Notably, significant heterogeneity was observed across the included studies, with an I^2^ value of 95% and a *p* value for heterogeneity of <0.001. This high degree of heterogeneity indicates considerable variability in effect estimates between studies, likely attributable to differences in study design, patient characteristics, indications for REBOA use, comparator treatments, and clinical protocols. Such heterogeneity underscores the need for cautious interpretation of the pooled results and suggests that the effect of REBOA on mortality may differ substantially across specific patient subgroups and clinical contexts.

### 3.4. Propensity Score–Matched and Non-Matched Analyses

**Figure 3:**
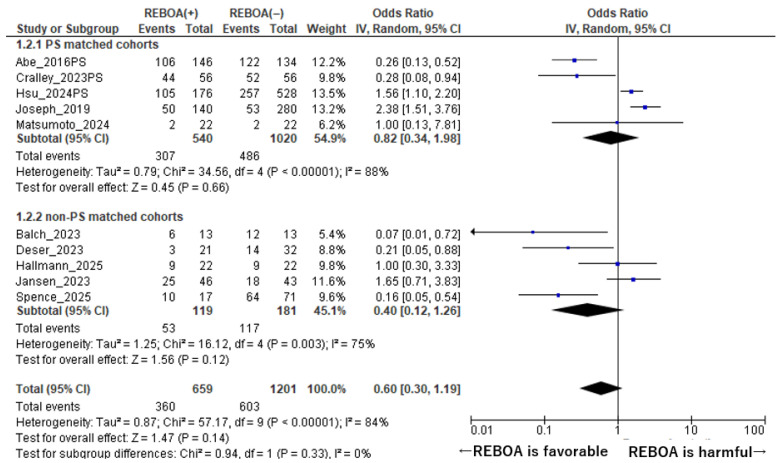
Forest Plot of Mortality Comparing REBOA and Non-REBOA Groups in Propensity Score–Matched and Non–Matched Studies. The forest plot was constructed using data from references [1,7,8,9,10,14,15,16,17,18].

A subgroup analysis stratified by the use of propensity score (PS) matching was performed to further assess the robustness of the association between REBOA use and mortality (Figure 3). Among the included studies, three reported outcomes for both propensity score–matched and non–propensity score–matched cohorts; for these studies, data derived from the propensity score–matched cohorts were preferentially used in the primary analyses to minimize the impact of baseline confounding [10,14,16]. In the propensity score–matched analysis, a total of five cohorts comprising 1560 patients were included [7,10,14,16,17]. Within these PS-matched cohorts, REBOA use was not associated with a statistically significant difference in mortality compared with non-REBOA management. The pooled odds ratio for mortality was 0.82 (95% CI: 0.34–1.98, *p* = 0.66), indicating no clear survival advantage or disadvantage associated with REBOA after adjustment for measured confounders. Substantial heterogeneity was observed among these studies (I^2^ = 88%, *p* for heterogeneity <0.001), suggesting considerable variability in effect estimates despite the use of PS matching. In contrast, the analysis of non–propensity score–matched cohorts included five cohorts with a total of 1860 patients [1,8,9,15,18]. In these unadjusted cohorts, the pooled odds ratio for mortality associated with REBOA use was 0.40 (95% CI: 0.12–1.26, *p* = 0.12). Although the point estimate favored REBOA, the confidence interval crossed unity, indicating no statistically significant difference in mortality between the REBOA and non-REBOA groups. Heterogeneity among the non-PS-matched cohorts was also substantial (I^2^ = 75%, *p* for heterogeneity <0.001), reflecting marked between-study variability. Taken together, these subgroup analyses demonstrate that the absence of a significant mortality benefit associated with REBOA persisted regardless of whether analyses were restricted to propensity score–matched cohorts or included unadjusted observational data, highlighting the consistency of the overall findings across different analytical approaches.

### 3.5. Geographic Differences: Western vs. Non-Western Countries

**Figure 4:**
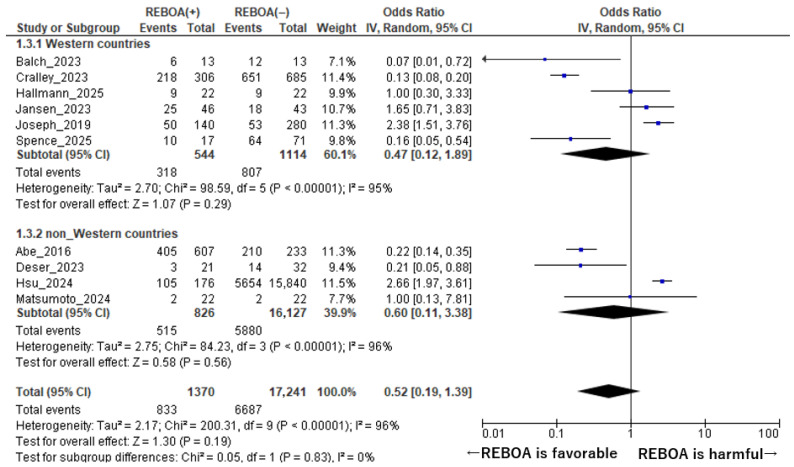
Forest Plot of Mortality Comparing REBOA and Non-REBOA Groups in Western and Non-Western Countries. The forest plot was constructed using data from references [1,7,8,9,10,14,15,16,17,18].

A subgroup analysis stratified by geographic region was performed to explore whether the association between REBOA use and mortality differed between Western and non-Western studies (Figure 4). This analysis was conducted to account for potential regional differences in patient characteristics, trauma systems, resource availability, and institutional experience with REBOA. As the majority of non-Western studies in this analysis were conducted in Japan and Taiwan (Asian settings), the geographic categorization effectively reflects an Asian versus non-Asian comparison; the term ‘non-Western’ was retained to align with established reporting conventions in the trauma literature. In studies conducted in Western countries, six studies comprising a total of 1658 patients were included [1,7,8,14,15,18]. In this subgroup, the pooled analysis demonstrated that REBOA use was not associated with a statistically significant reduction in mortality compared with non-REBOA management. The pooled odds ratio for mortality was 0.47 (95% CI: 0.12–1.89, *p* = 0.29), indicating no clear survival benefit. Substantial heterogeneity was observed among the Western studies, with an I^2^ value of 95% and a *p* value for heterogeneity of <0.001, suggesting considerable variability in effect estimates across studies. Similarly, in non-Western studies, four studies encompassing a much larger combined population of 18,067 patients were included in the analysis [9,10,16,17]. In this subgroup, REBOA use was also not associated with a significant difference in mortality when compared with non-REBOA management. The pooled odds ratio was 0.60 (95% CI: 0.11–3.38, *p* = 0.56), with wide confidence intervals reflecting substantial uncertainty in the estimated effect size. Heterogeneity among the non-Western studies was likewise pronounced (I^2^ = 96%, *p* for heterogeneity <0.001).

Overall, these findings indicate that the absence of a statistically significant mortality benefit associated with REBOA was consistent across geographic regions. Despite differences in healthcare systems, trauma care infrastructure, and clinical practice patterns between Western and non-Western settings, no regional subgroup demonstrated a clear survival advantage with REBOA use. The high degree of heterogeneity observed in both subgroups underscores the need for cautious interpretation of these results and suggests that regional factors alone do not adequately explain the variability in reported outcomes.

### 3.6. Mortality in Trauma vs. Non-Trauma Patients

**Figure 5:**
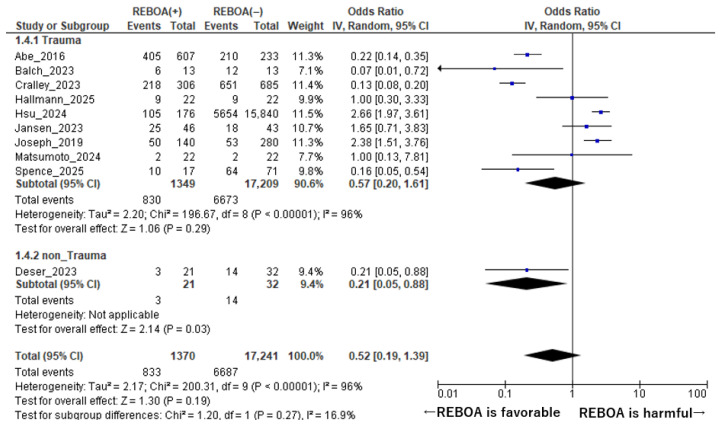
Forest Plot of Mortality Comparing REBOA and Non-REBOA Groups in Trauma vs. Non-Trauma Patients. The forest plot was constructed using data from references [1,7,8,9,10,14,15,16,17,18].

When stratified by clinical setting, distinct differences were observed in the association between REBOA use and mortality (Figure 5). Among trauma patients, nine studies comprising a total of 18,558 patients were included in the analysis [1,7,8,10,14,15,16,17,18]. In this subgroup, REBOA use was not associated with a statistically significant reduction in mortality compared with non-REBOA management. The pooled odds ratio for mortality was 0.57 (95% CI: 0.20–1.61, *p* = 0.29), indicating no clear survival benefit in the trauma population. Notably, substantial heterogeneity was observed across the trauma studies (I^2^ = 96%, *p* for heterogeneity <0.001), reflecting marked variability in effect estimates that may be attributable to differences in injury mechanisms, severity of hemorrhage, timing of intervention, and institutional protocols for REBOA deployment.

In contrast, the analysis of non-trauma patients demonstrated a different pattern. Only one study, including 53 patients, met the inclusion criteria for this subgroup [9]. In this non-trauma cohort, REBOA use was associated with a significantly lower mortality compared with non-REBOA management, with a pooled odds ratio of 0.21 (95% CI: 0.05–0.88, *p* = 0.03). Although this finding suggests a potential survival benefit of REBOA in non-traumatic hemorrhagic conditions, the result is based on a single, relatively small study (n = 53) and should therefore be interpreted with caution. This finding is hypothesis-generating only and should not be used to support definitive clinical recommendations; larger prospective studies are required to confirm any potential benefit of REBOA in non-traumatic hemorrhage.

Overall, these subgroup analyses indicate that the effect of REBOA on mortality differs according to clinical setting. While no significant benefit was observed among trauma patients, a potential advantage was suggested in non-trauma patients. However, the limited number of studies and patients in the non-trauma subgroup underscores the need for additional high-quality investigations to better define the role of REBOA in specific non-traumatic clinical contexts.

## 4. Discussion

The lack of mortality improvement in trauma patients demonstrated in this study aligns with and reinforces the conclusions of the landmark randomized controlled trial in this field, the UK-REBOA trial [8]. This first-of-its-kind RCT suggested that a strategy of using REBOA with standard care, compared to standard care alone, did not reduce 90-day mortality and might even increase it (54% vs. 42%; OR 1.58). The posterior probability that REBOA was harmful (OR > 1) reached 86.9%. Although our meta-analysis is composed of observational studies, its large-scale pooled results provide real-world evidence suggesting that the concerns raised by the UK-REBOA trial are not idiosyncratic but reflect a broader reality in trauma care. In our pooled analysis, REBOA was not associated with a significant reduction in mortality in trauma patients (OR 0.57, 95% CI: 0.20–1.61, *p* = 0.29), consistent with the UK-REBOA trial findings.

The reasons for this lack of benefit, or potential harm, are likely multifactorial, as reflected in the details of the UK-REBOA trial. First is the delay to definitive hemostasis. In that trial, the median time to definitive hemorrhage control was 19 min longer in the REBOA group. REBOA is merely a “bridge” to definitive treatment, and numerous studies have emphasized that any delay to definitive interventions like surgery or angioembolization can be fatal [19]. Second is the issue of ischemic burden. Complete aortic occlusion causes severe distal ischemia, metabolic acidosis, and reperfusion injury, which can contribute to multiple organ failure and death. The patients in the UK-REBOA trial had very long pre-hospital times (median >90 min) and were already in a state of profound and prolonged shock before REBOA was placed, which likely exacerbated the additional ischemic insult from REBOA. Third are the procedural challenges. While the safety and feasibility of REBOA have been cautiously evaluated since its early adoption [20], the UK-REBOA trial reported protocol violations and instances where the balloon was not fully inflated in many patients in the REBOA group, highlighting the practical difficulties of securing arterial access and rapidly deploying the device in a patient in circulatory collapse. Furthermore, the large-caliber sheaths used for the procedure can contribute to access site complications such as vascular injury, leading to efforts to reduce complications by using smaller devices [21].

The timeliness of our study represents an important strength, allowing for a more comprehensive assessment of REBOA in trauma compared with previous research. Notably, two recent high-profile syntheses have further advanced this field: Castellini et al. performed a systematic review and meta-analysis of 11 studies, demonstrating that REBOA was associated with lower mortality compared with resuscitative thoracotomy, while no significant benefit was observed versus non-REBOA management [22], and Manzano-Nunez R et al. conducted a systematic review and meta-analysis including 25 studies, reporting that REBOA was associated with lower in-hospital mortality compared with resuscitative thoracotomy, while no significant survival benefit was observed compared with non-REBOA management [23]—the most comprehensive synthesis to date. Our review complements these publications by specifically examining trauma versus non-trauma subgroup effects and geographic variation, analytical dimensions not fully addressed in prior guidelines. For example, a 2021 meta-analysis by Castellini et al. concluded that there was no significant difference in mortality when comparing REBOA with non-REBOA interventions [22]. However, that analysis suggested a possible benefit of REBOA when compared with resuscitative thoracotomy (RT). This potential advantage of REBOA over RT has been suggested in several meta-analyses published prior to our study [24]. This observation is further supported by animal studies indicating that REBOA may maintain more favorable hemodynamics and impose a lower metabolic burden than RT [25]. Together, these findings reflect the clinical trend toward considering REBOA as a less invasive alternative to RT [26]. In addition, some previous meta-analyses included overlapping sub-analyses derived from the same patient cohorts; such overlap was excluded in the present study.

Furthermore, the term “trauma” encompasses a highly heterogeneous patient population, and the neutral overall result observed in our study may obscure potential benefits in specific subgroups. In addition to differences in injury patterns, the severity of hemorrhagic shock at presentation may also substantially influence the effectiveness of REBOA. Several observational studies have suggested that the potential survival benefit of REBOA may be most pronounced in patients who are in extremis or experiencing traumatic cardiac arrest, where rapid aortic occlusion may temporarily restore central perfusion and provide time for definitive hemorrhage control. In contrast, in patients with severe shock but not in extremis, traditional damage control surgery or other definitive hemostatic strategies may remain more appropriate. Previous studies have suggested a possible benefit of REBOA in patients with isolated pelvic fracture trauma [27], whereas others have reported less favorable outcomes in cases of high-grade liver injury or penetrating abdominal vascular injury [28,29]. The role of REBOA in traumatic brain injury also remains uncertain, with some reports suggesting potential advantages over RT and others advocating cautious application [30,31]. A recent study examined the safety of REBOA in patients with concomitant severe TBI using propensity score matching to address this clinically important question [31]. Collectively, these findings suggest that, if REBOA has a role in trauma care, it is likely as a selective intervention limited to carefully chosen injury patterns—where the bleeding source is anatomically amenable to occlusion and the risk of exacerbating associated injuries such as TBI is relatively low—rather than as a uniform treatment for hemorrhagic shock. This need for subgroup-specific consideration extends to pediatric trauma, in which physiological characteristics and anatomical size differ substantially from those of adults, and prior studies have described the practice patterns and challenges of REBOA use in this population [32]. More recently, efforts have also been made to explore expanded indications by applying REBOA to selected patients with penetrating thoracic trauma and proposing deployment algorithms for this purpose [33]. One of the clinically relevant findings of our study is the observation of a substantial reduction in mortality associated with REBOA in non-trauma patients. To interpret this finding, it is necessary to consider the pathophysiological differences between traumatic and non-traumatic hemorrhage, as well as the distinct clinical contexts in which REBOA is applied.

Taken together, the present findings suggest that the clinical impact of REBOA is highly context dependent and closely related to patient selection and clinical setting. In an unselected trauma population with hemorrhagic shock, the currently available evidence does not demonstrate a clear mortality benefit associated with REBOA use (overall pooled OR 0.52, 95% CI: 0.19–1.39, *p* = 0.19; trauma subgroup OR 0.57, 95% CI: 0.20–1.61, *p* = 0.29). This observation is consistent with high-level contemporary evidence, including the UK-REBOA trial, and supports a cautious interpretation of REBOA as a broadly applicable intervention in trauma care. The absence of benefit in this setting likely reflects the marked heterogeneity inherent to traumatic hemorrhage, where variability in injury mechanisms, physiological severity, timing of intervention, and institutional experience may substantially influence outcomes. In such complex clinical scenarios, the theoretical advantages of proximal aortic occlusion may be offset by delays to definitive hemostasis, ischemic burden, and procedural challenges. In contrast, our findings indicate that REBOA may be associated with reduced mortality in patients with non-traumatic hemorrhage (OR 0.21, 95% CI: 0.05–0.88, *p* = 0.03, based on a single study, n = 53). This finding is exploratory and hypothesis-generating; it should not be used to support clinical recommendations without further confirmatory evidence. These results suggest that, when applied in clinical contexts with a clearly identified bleeding source and a well-defined therapeutic goal, REBOA may function effectively as a temporizing measure. Non-traumatic emergencies such as ruptured abdominal aortic aneurysm or severe postpartum hemorrhage represent settings in which REBOA can be deployed in a controlled environment, often as a proactive bridge to definitive hemostasis. In these situations, the physiological rationale for aortic occlusion is more straightforward, and competing factors commonly encountered in trauma—such as multiple injury sites or concomitant traumatic brain injury—are less prominent. Nevertheless, the current evidence supporting REBOA use in non-trauma populations remains limited, and these findings should be interpreted with appropriate caution. These observations have important implications for future research. First, further large-scale studies enrolling broadly defined trauma shock populations are unlikely to provide substantial incremental insight and may continue to be limited by heterogeneity and residual confounding. Instead, future investigations should adopt a more targeted approach, focusing on carefully defined patient phenotypes in whom the potential benefits of REBOA are most plausible. Second, high-quality prospective studies, and where feasible randomized controlled trials, are needed to more definitively assess the role of REBOA in specific non-traumatic settings, such as its adjunctive use during endovascular repair for ruptured abdominal aortic aneurysm. Third, given ongoing concerns regarding distal ischemia and related complications, research priorities should include the evaluation of strategies designed to mitigate ischemic burden. In this regard, partial REBOA (pREBOA) has emerged as a promising technique; however, its clinical effectiveness, optimal perfusion targets, and standardized protocols remain insufficiently defined. Clarifying these aspects represents a critical next step toward refining the selective and evidence-based use of REBOA in contemporary clinical practice.

## 5. Study Limitations

Several limitations should be considered when interpreting the findings of this study. First, the majority of the included studies were retrospective observational in design, which are inherently susceptible to selection bias and residual confounding. Although we performed multiple subgroup analyses, including analyses restricted to propensity score–matched cohorts, these approaches cannot fully eliminate bias arising from unmeasured or inadequately captured confounders. In particular, clinically important factors such as the appropriateness of REBOA indication, the timing of balloon deployment relative to hemorrhage control or resuscitation, and institutional experience with REBOA could not be consistently accounted for across studies. Formal meta-regression incorporating key covariates such as injury severity score, time to balloon deployment, or occlusion zone was not feasible due to inconsistent reporting of these parameters across included studies; this represents an important limitation and a priority area for future research. Second, there was substantial clinical and methodological heterogeneity among the included studies. This heterogeneity encompassed differences in patient populations (e.g., trauma versus non-trauma cases, blunt versus penetrating mechanisms of injury), variations in REBOA protocols (including occlusion zone, duration of balloon inflation, and the use of partial or intermittent REBOA), as well as differences in comparator management strategies and definitions of outcomes. Furthermore, patients in both the REBOA and non-REBOA groups were managed according to standard institutional protocols, leading to inherent practice variability that could not be fully controlled for. Such heterogeneity may have influenced the magnitude and direction of the pooled effect estimates and complicates direct comparisons across studies. Additionally, the inclusion of a single RCT (UK-REBOA trial, n = 89) alongside predominantly large retrospective observational studies warrants consideration. Formal pooling of RCT and observational data under the same random-effects model has inherent methodological limitations; however, the directional alignment between the RCT result and the overall pooled estimate (no mortality benefit in trauma patients) provides reassurance regarding consistency of findings. Consequently, while pooled analyses provide an overall summary of available evidence, this heterogeneity limits the interpretability of the results and precludes definitive conclusions regarding optimal patient selection, timing, and procedural strategies for REBOA use in diverse clinical settings. Third, although funnel plot asymmetry and Egger’s regression test (z = 2.17, *p* = 0.03) suggested potential publication bias, these assessments have limited reliability with fewer than 10 studies and should be interpreted with caution. Formal correction methods such as trim-and-fill analysis were not performed due to the small number of included studies, and this potential publication bias may have influenced the pooled estimates. Fourth, the inability to account for technical factors including occlusion zone (Zone I vs. Zone III), duration of balloon inflation, and the use of partial versus complete REBOA represents a critical gap, as these factors are known to significantly influence outcomes and could not be controlled for due to inconsistent reporting in the primary studies.

## 6. Conclusions

REBOA was not associated with improved overall survival, particularly in unselected trauma populations. While a potential benefit was observed in a non-trauma cohort, this finding is based on a single small study and must be interpreted cautiously as exploratory. These results highlight the importance of patient selection and suggest that REBOA should be applied selectively rather than routinely in hemorrhagic shock. Further large-scale prospective studies are needed to define its optimal clinical role.

## Figures and Tables

**Table 1:** Characteristics of included studies.

Author (Year)	Country of 1st Author	Sample Size (REBOA vs. No-REBOA)	Age	Female, n (%)	Target Disease	Study Design	PS Matching (If Retrospective)	Outcome	Study Period
Abe T 2016 [10]	Japan	840 (607 vs. 233)	Not reported	Not reported	Trauma	Retrospective study	Yes	In-hospital mortality	2004–2013
Balch J 2023 [1]	USA	26 (13 vs. 13)	40 (27–63)	6 (23%)	Trauma	Retrospective study	No	In-hospital mortality	August 2015–November 2019
Cralley A 2023 [14]	USA	991 (306 vs. 685)	32 (25–48)	183 (18.5%)	Trauma	Retrospective study	Yes	Mortality	October 2013–September 2021
Deser S 2024 [9]	Turkey	53 (21 vs. 32)	71.9 (51–89)	7 (13.2%)	Ruptured abdominal aortic aneurysm	Retrospective study	No	30-day mortality	January 2014–November 2021
Hallmann B 2025 [15]	Austria	44 (22 vs. 22)	55 (42–64)	Not reported	Trauma	Retrospective study	No	30-day mortality	January 2019–October 2023
Hsu C 2024 [16]	Taiwan	16,016 (176 vs. 15,840)	53 (33–68)	5400 (33.7%)	Trauma with traumatic brain injury	Retrospective study	Yes	Mortality	2017–2019
Jansen J 2023 [8]	UK	89 (46 vs. 43)	Not reported	Not reported	Trauma	Randomized controlled trial	Not applicable	90-day mortality	October 2017–March 2022
Joseph B 2019 [7]	USA	420 (140 vs. 280)	43.3	113 (26.9%)	Trauma	Retrospective study	Yes (PS-only data)	Mortality	2015–2016
Matsumoto S 2024 [17]	Japan	44 (22 vs. 22)	44.5 (26.5–53.5)	11 (25%)	Trauma	Retrospective study	Yes (PS-only data)	In-hospital mortality	2008–2019
Spence S 2025 [18]	USA	88 (17 vs. 71)	Not reported	Not reported	Trauma	Retrospective study	No	Mortality	January 2012–October 2022

Values are presented as number, number (percentage), or median [interquartile range], as reported in each study. Sample size is shown as total number of patients with the distribution between the REBOA and no-REBOA groups. Age and sex data were unavailable or incompletely reported in some studies and are indicated as “Not reported.” For retrospective studies, the use of propensity score (PS) matching is specified; “PS-only data” indicates studies in which only propensity score–matched cohorts were available and unmatched data were not reported. Outcomes are reported as defined in the original studies, including in-hospital mortality, 30-day mortality, 90-day mortality, or overall mortality. REBOA, resuscitative endovascular balloon occlusion of the aorta; PS, propensity score.

## Data Availability

The datasets generated and/or analyzed during the current study are available from the corresponding author upon reasonable request.

## Supplementary Materials

The following supporting information can be downloaded at: https://www.mdpi.com/article/10.3390/diseases14040122/s1, Supplemental material 1: Literature search strategy for REBOA systematic review, Supplemental material 2: Risk of Bias Assessment Using the Newcastle–Ottawa Scale (NOS); Supplemental material 3: Funnel plot for assessment of publication bias.

## Abbreviations

AAST	American Association for the Surgery of Trauma
CI	Confidence interval
NCTH	Non-compressible torso hemorrhage
NOS	Newcastle–Ottawa Scale
OR	Odds ratio
PS	Propensity score
rAAA	Ruptured abdominal aortic aneurysm
RCT	Randomized controlled trial
REBOA	Resuscitative endovascular balloon occlusion of the aorta
RT	Resuscitative thoracotomy
RT-ACC	Resuscitative thoracotomy with aortic cross-clamping
TBI	Traumatic brain injury
UMIN	University Hospital Medical Information Network
