# Supplementary material for: Impact of Resuscitative Endovascular Balloon Occlusion of the Aorta on In-Hospital and Short-Term Mortality: A Systematic Review and Meta-Analysis
Source: Diseases. 2026 Mar 27;14(4):122. doi: 10.3390/diseases14040122 (PMC13115052; doi:10.3390/diseases14040122)
Supplement: Supplementary file 1 [file diseases-14-00122-s001.zip › Supplemental material.pdf]

Supplemental material 1. Literature search strategy for REBOA systematic review

・Pubmed 266 (27th/Oct/2025)

(("resuscitative endovascular balloon occlusion of the aorta"[Title/Abstract]
OR "REBOA"[Title/Abstract]
OR ("endovascular balloon occlusion"[Title/Abstract] AND "aorta"[Title/Abstract])
OR "aortic balloon occlusion"[Title/Abstract]
OR "endovascular aortic occlusion"[Title/Abstract])
AND
("mortality"[MeSH Terms]
OR "survival"[MeSH Terms]
OR "death"[Title/Abstract]
OR "outcome"[Title/Abstract]
OR "prognosis"[Title/Abstract]
OR "surviv*"[Title/Abstract])
AND
("trauma"[MeSH Terms]
OR "injuries"[MeSH Terms]
OR "non-trauma"[Title/Abstract]
OR "hemorrhagic shock"[Title/Abstract]
OR "hemorrhage"[Title/Abstract]
OR "bleeding"[Title/Abstract]
OR "ruptured abdominal aortic aneurysm"[Title/Abstract]
OR "postpartum hemorrhage"[Title/Abstract]))
NOT
(animals[mh] NOT humans[mh])

・WOS 573 (27th/Oct/2025)

TS=("resuscitative endovascular balloon occlusion of the aorta"
OR REBOA
OR ("endovascular balloon occlusion" NEAR/3 aorta)
OR "aortic balloon occlusion"
OR "endovascular aortic occlusion")
AND
TS=(mortality OR survival OR prognosis OR outcome OR death OR surviv*)
AND
TS=(trauma OR "hemorrhagic shock" OR hemorrhage OR bleeding OR "ruptured abdominal aortic aneurysm" OR "postpartum hemorrhage" OR non-trauma)
NOT
TS=(animal*)

・Cochrane Library 32 (27th/Oct/2025)

("resuscitative endovascular balloon occlusion of the aorta"
OR REBOA
OR ("endovascular balloon occlusion" NEXT aorta)
OR "aortic balloon occlusion"
OR "endovascular aortic occlusion")
:ti,ab,kw
AND
(mortality OR survival OR prognosis OR outcome OR death OR surviv*):ti,ab,kw
AND
(trauma OR "hemorrhagic shock" OR hemorrhage OR bleeding OR "ruptured abdominal aortic aneurysm" OR "postpartum hemorrhage" OR non-trauma):ti,ab,kw

Supplemental material 2. Risk of Bias Assessment Using the Newcastle–Ottawa Scale (NOS)

Study	Selection (0–4)	Comparability (0–2)	Outcome (0–3)	Total Score	Quality
Abe T (2016)	4	2	3	9	High
Balch J (2023)	3	0	2	5	Moderate
Cralley A (2023)	4	2	3	9	High
Deser S (2024)	3	0	2	5	Moderate
Hallmann B (2025)	3	0	2	5	Moderate
Hsu C (2024)	4	2	3	9	High
Joseph B (2019)	4	2	3	9	High
Matsumoto S (2024)	4	2	3	9	High
Spence S (2025)	3	0	2	5	Moderate

The methodological quality of observational studies was assessed using the Newcastle–Ottawa Scale (NOS), which evaluates studies across three domains: selection, comparability, and outcome. Studies scoring 7–9 points were considered high quality, 4–6 points moderate quality, and ≤3 points low quality. The randomized controlled trial was excluded from NOS assessment.

Supplemental material 3. Funnel plot for assessment of publication bias. Funnel plot of individual study effect sizes (odds ratios) plotted against the standard error of the log-transformed odds ratio (SE[log(OR)]). Each open circle represents an individual study. The vertical dashed line indicates the pooled effect estimate from the random-effects meta-analysis. Asymmetry of the funnel plot suggests potential small-study effects or publication bias [Egger's regression test ($z = 2.17$, $p = 0.03$)].

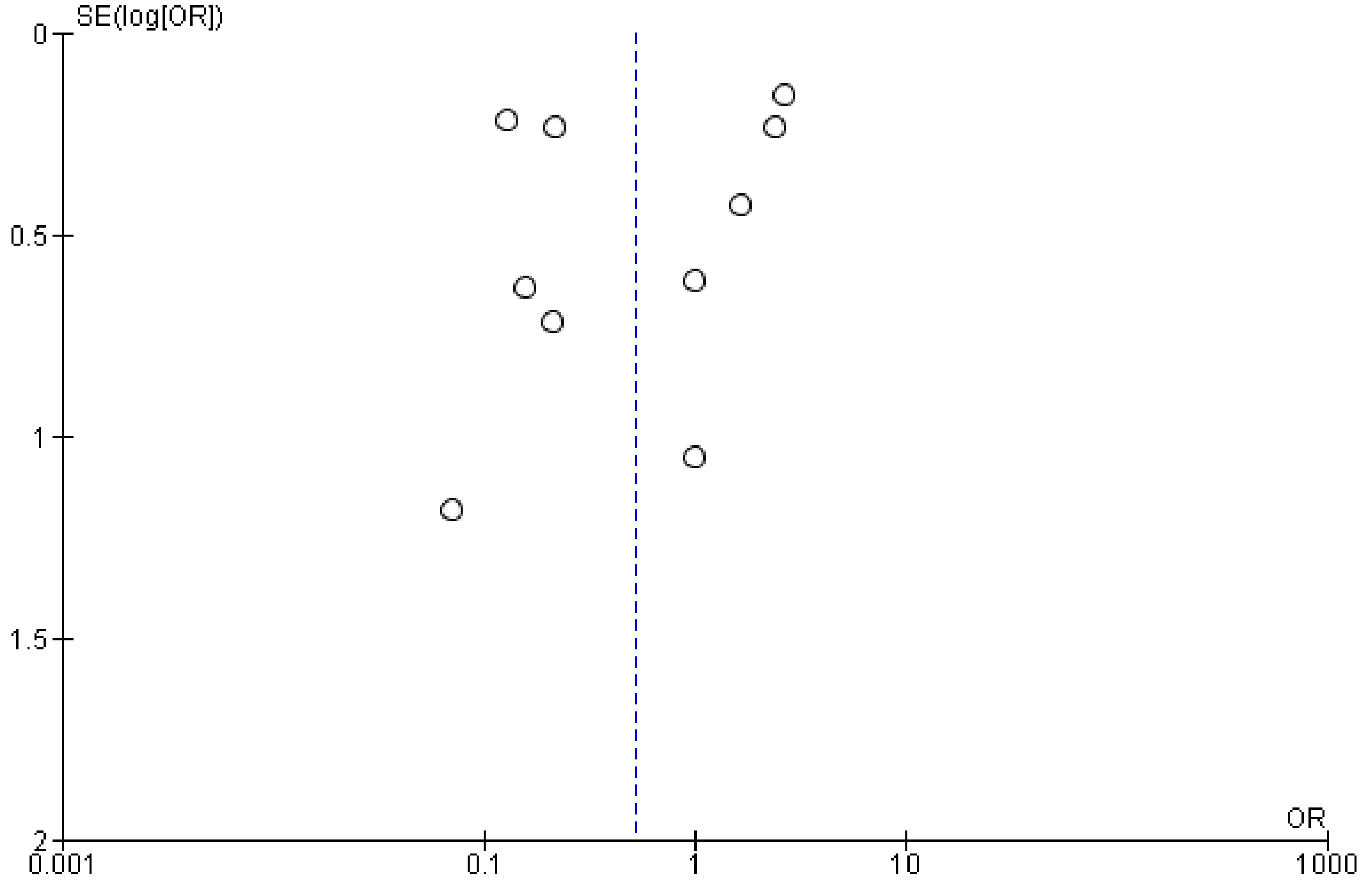

Supplemental material 3. Funnel plot for assessment of publication bias. Funnel plot of individual study effect sizes (odds ratios) plotted against the standard error of the log-transformed odds ratio (SE[log(OR)]). Each open circle represents an individual study. The vertical dashed line indicates the pooled effect estimate from the random-effects meta-analysis. Asymmetry of the funnel plot suggests potential small-study effects or publication bias [Egger's regression test ($z = 2.17$, $p = 0.03$)].
