# Supplementary figures and images for: Impact of Resuscitative Endovascular Balloon Occlusion of the Aorta on In-Hospital and Short-Term Mortality: A Systematic Review and Meta-Analysis
Source: Diseases. 2026 Mar 27;14(4):122. doi: 10.3390/diseases14040122 (PMC13115052; doi:10.3390/diseases14040122)

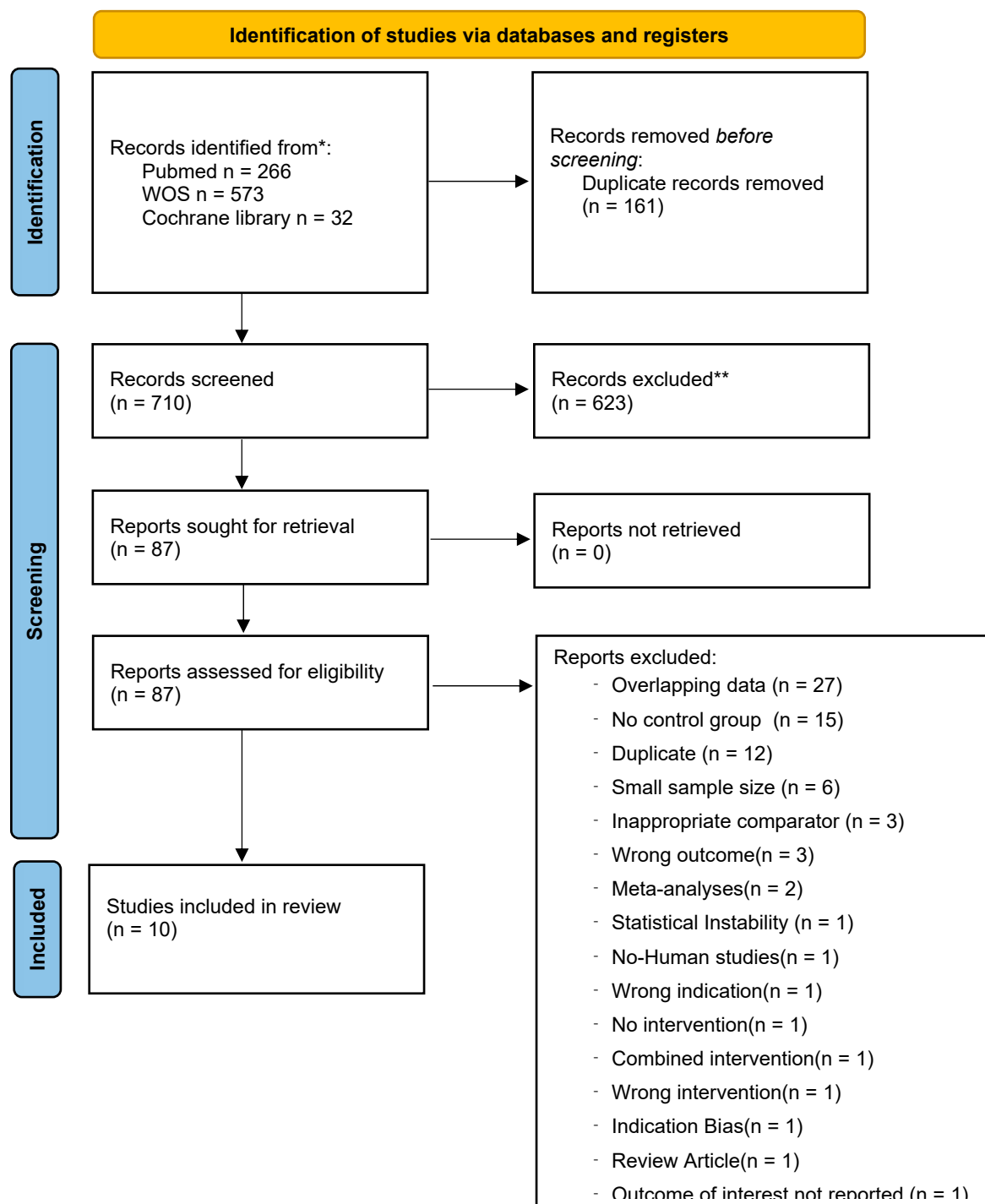

Supplement: Supplementary file 1 [file diseases-14-00122-s001.zip › PRISMA_2020_flow_diagram_REBOA.pdf]
